# An AIREless Breath: Pneumonitis Caused by Impaired Central Immune Tolerance

**DOI:** 10.3389/fimmu.2020.609253

**Published:** 2021-01-27

**Authors:** Elise M. N. Ferré, Michail S. Lionakis

**Affiliations:** Fungal Pathogenesis Section, Laboratory of Clinical Immunology and Microbiology, National Institute of Allergy and Infectious Diseases, National Institutes of Health, Bethesda, MD, United States

**Keywords:** Autoimmune-polyendocrinopathy-candidiasis-ectodermal dystrophy (APECED), autoimmune polyglandular syndrome type-1 (APS-1), autoimmune regulator (AIRE), pneumonitis, interstitial lung disease, bronchiectasis

## Abstract

Autoimmune-polyendocrinopathy-candidiasis-ectodermal dystrophy (APECED), a monogenic disorder caused by biallelic mutations in the *AIRE* gene, has historically been defined by the development of chronic mucocutaneous candidiasis together with autoimmune endocrinopathies, primarily hypoparathyroidism and adrenal insufficiency. Recent work has drawn attention to the development of life-threatening non-endocrine manifestations such as autoimmune pneumonitis, which has previously been poorly recognized and under-reported. In this review, we present the clinical, radiographic, autoantibody, and pulmonary function abnormalities associated with APECED pneumonitis, we highlight the cellular and molecular basis of the autoimmune attack in the AIRE-deficient lung, and we provide a diagnostic and a therapeutic roadmap for patients with APECED pneumonitis. Beyond APECED, we discuss the relevance and potential broader applicability of these findings to other interstitial lung diseases seen in secondary AIRE deficiency states such as thymoma and RAG deficiency or in common polygenic autoimmune disorders such as idiopathic Sjögren’s syndrome.

## Introduction

Autoimmune-polyendocrinopathy-candidiasis-ectodermal dystrophy (APECED), also known as autoimmune polyglandular syndrome type-1 (APS-1), is a rare disorder resulting from biallelic mutations in the autoimmune regulator (*AIRE*) gene. AIRE is a thymus-enriched transcription regulator integral for enforcing central immune tolerance. AIRE-deficiency leads to multiorgan system autoimmunity and susceptibility to chronic mucocutaneous candidiasis (CMC). Diagnosis relies on developing two (“diagnostic dyad”) out of any three “classic triad” manifestations of CMC, hypoparathyroidism, and adrenal insufficiency. Development of a diagnostic dyad raises suspicion for APECED, which is then confirmed by *AIRE* gene sequencing. Detection of type I interferon (IFN-α/IFN-ω) autoantibodies is sensitive and specific for APECED and is useful for diagnosis (1). While the classic triad is quite characteristic for APECED, exclusive reliance on the classic triad manifestations results in delayed clinical diagnosis as a variety of non-triad non-endocrine manifestations develop often before reaching a classic diagnostic dyad (2). To that end, we have proposed inclusion of an adjunct triad of early-onset manifestations, namely APECED rash, intestinal dysfunction, and enamel hypoplasia, into expanded diagnostic criteria which would reduce the time to clinical diagnosis by half (3). Establishing an earlier diagnosis is important as it can enable screening for life-threatening endocrinopathies and prompt recognition and treatment of non-endocrine autoimmune manifestations such as hepatitis (4) or pneumonitis (5).

With regard to pneumonitis, prior studies had suggested it to be an uncommon manifestation of APECED (prevalence in all previously-published work, ~2%). A small number of affected patients (2.7–4.5%) had been described among Turkish, Russian, and Indian APECED cohorts. Importantly, the foundational APECED cohort descriptions in Finns, Sardinians, or Iranian Jews do not highlight pneumonitis nor is it a prominent feature in the literature among APECED patients from the British Isles (6–25). In contrast, in a prospective observational natural history study at the NIH, we diagnosed >40% of consecutively-enrolled APECED patients with autoimmune pneumonitis; notably, pneumonitis symptoms presented early in life, often before developing a classic diagnostic dyad (5).

## Definition and Clinical Presentation of Autoimmune-Polyendocrinopathy-Candidiasis-Ectodermal Dystrophy Pneumonitis

APECED pneumonitis presents clinically with chronic respiratory symptoms lasting >4 weeks with accompanying radiographic abnormalities of interstitial lung disease (ILD) and/or bronchiectasis. Affected patients most commonly present with daily cough with or without sputum production, and frequently report nocturnal bouts of cough (60%) awakening them from sleep. Less frequently, dyspnea on exertion (57%), pleuritic chest pain (48%), wheezing (43%), and fevers (29%) occur (5). Importantly, a small proportion of patients (<5–10%) is asymptomatic early in the course of pneumonitis (5).

Non-contrast computed tomography (CT) of the chest reveals abnormalities consistent with ILD and/or bronchiectasis. Specifically, ground-glass opacities (GGO) or mosaicism and bronchiectasis are the most common abnormalities; they are seen, either alone or in combination, in all patients with APECED pneumonitis, including those without respiratory symptoms and negative lung-targeted autoantibodies (see below) (5). Additional less common radiographic findings include a tree-in-bud pattern, nodular opacities, and mucus plugging. Taken together, non-contrast chest CT imaging is the most sensitive screening tool for APECED pneumonitis.

In keeping with these chronic symptoms and radiographic abnormalities, APECED pneumonitis leads to abnormal pulmonary function (5, 26, 27). Indeed, affected patients display decreased diffusing capacity of the lungs for carbon monoxide with or without a ventilatory defect by spirometry presenting as obstructive, restrictive, or a mixed pattern of both. A 6 min walk test typically shows decreased walk distance and oxygen desaturation (5).

### Progression of Untreated Pneumonitis Causes Morbidity and Mortality

**Figure 1:**
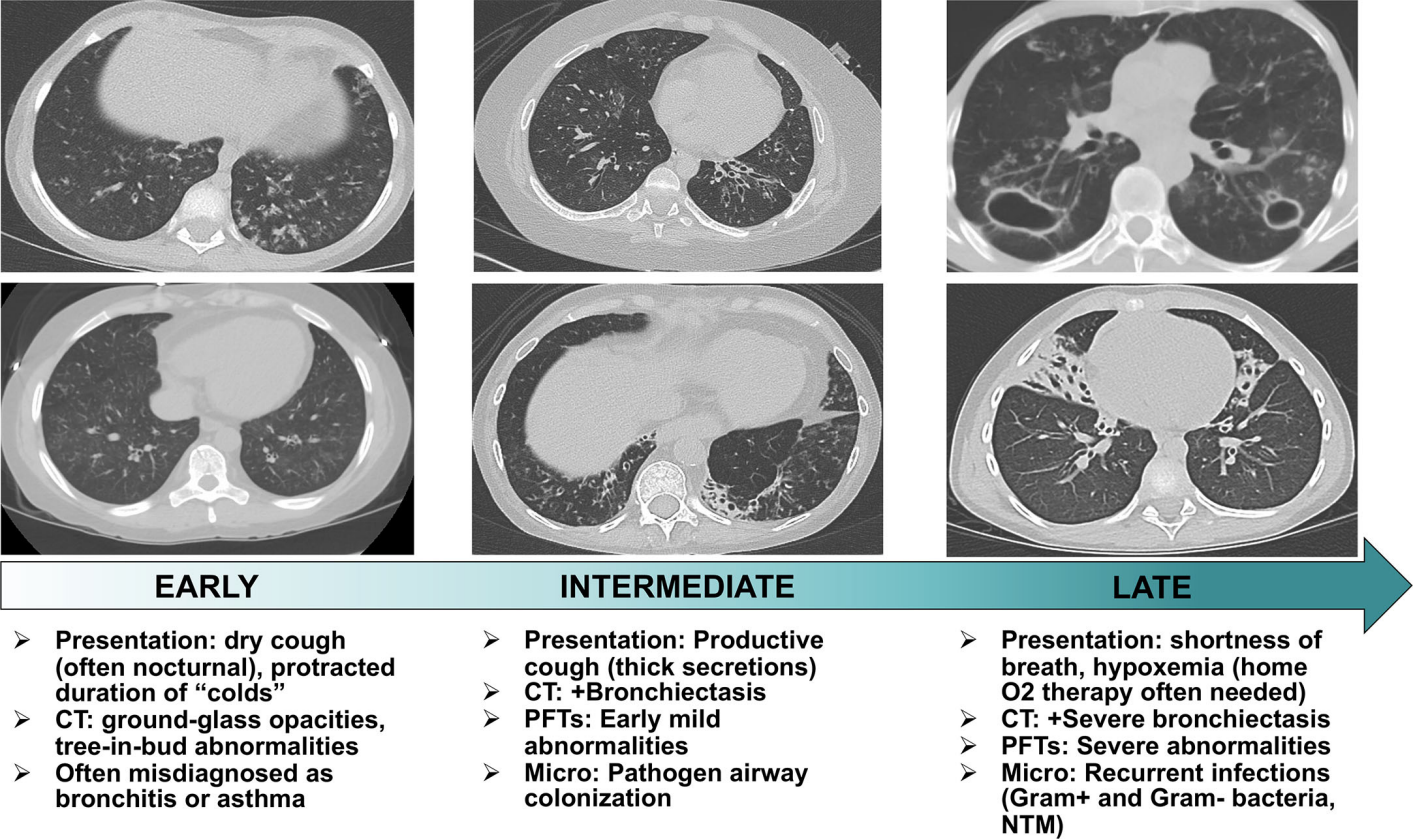
Stages of temporal progression of APECED-associated pneumonitis. Flow chart summarizing the temporal progression of symptoms, radiographic and pulmonary function test abnormalities, and microbiological findings in patients with APECED pneumonitis. CT, computed tomography; PFT, pulmonary function tests; Micro, microbiological findings; NTM, nontuberculous mycobacteria.

Through the course of our study, we encountered patients across the spectrum of pneumonitis severity which allowed us to characterize the temporal progression of clinical and radiographic features of APECED pneumonitis. Early-stage disease manifests with dry cough associated with GGO and/or a tree-in-bud pattern without bronchiectasis (**Figure 1**). Without immunosuppression, pneumonitis progresses to bronchiectasis-associated structural lung disease presenting with productive cough and bacterial airway colonization. Late-stage untreated pneumonitis features progressively worsening bronchiectasis-associated structural lung disease with development of recurrent infections by Gram-negative bacteria, Gram-positive bacteria, or nontuberculous mycobacteria (NTM) leading to hypoxemia requiring home oxygen therapy (5).

The few clinical cases previously described in the literature corroborate our study observations. DeLuca et al. and Alimohammadi et al. reported a Sicilian child who first developed productive cough and recurrent lower respiratory tract infections at the age of 5 years. The patient’s pneumonitis progressed over time with development of a severe obstructive defect, bronchiectasis, chronic airway colonization with *Burkholderia*, and hypoxemia requiring daily oxygen supplementation at the age of 14 years. The patient succumbed to pneumonitis complications when 18 years-old (26, 27). Alimohammadi and colleagues described three additional patients who developed chronic cough in childhood and progressed clinically with recurrent lower respiratory tract infections, an obstructive ventilatory defect, and radiographic evidence of bronchiectasis and/or GGO. One of the patients was oxygen-dependent by 19 years and another died at 37 years from respiratory failure (27).

Therefore, disease progression from symptom onset to end-stage lung disease is highly variable as demonstrated by the aforementioned cases. Similarly, in our recent study we reported a 54-year-old man who developed chronic cough when 5 years-old and progressed over 40 years to eventually develop cavitary pulmonary NTM infection complicated by bronchopulmonary fistula and empyema, chronic hypoxemia requiring daily supplemental oxygen, and death at 56 years. His case stands in contrast to a 14-year-old boy who rapidly progressed from cough onset at 7 years to home oxygen therapy at 11 years and death at 14 years (5).

Therefore, timely diagnosis is necessary to ensure early initiation of immunomodulation in order to arrest progression to bronchiectasis-associated structural lung disease. However, this can be challenging to achieve as symptoms frequently begin in early life and often before the patient develops a classic diagnostic dyad that would raise suspicion for APECED. Even patients with confirmed APECED typically experience delays in pneumonitis diagnosis due to the poor characterization of the entity in the previously-published literature. Consequently, patients are often misdiagnosed with asthma or bronchitis resulting in treatment delays thereby increasing the risk of developing structural lung disease and associated morbidity and mortality. For this reason, we recommend that all APECED patients, regardless of symptoms, undergo periodic screening with chest CT to achieve early diagnosis of APECED pneumonitis (5). Moreover, a high index of suspicion for APECED is required by pediatricians and pulmonologists in children who develop chronic respiratory symptoms in the setting of CMC and/or autoimmune manifestations within the classic and/or adjunct diagnostic criteria of APECED.

### Pathogenesis of Autoimmune-Polyendocrinopathy-Candidiasis-Ectodermal Dystrophy Pneumonitis

#### *AIRE* Genetics and Non-AIRE Modifiers may Impact Pneumonitis Prevalence

APECED is caused by biallelic *AIRE* mutations (28, 29). In our genotype-phenotype analysis, we found an association between carrying the c.967_979del13 mutation in homozygosity with decreased time to development of pneumonitis (5). Autosomal dominant (AD) *AIRE* mutations in the first plant homeodomain (PHD1) zinc finger domain and in the SAND domain have been described to cause organ-specific autoimmune disease resulting in milder phenotypes with reduced penetrance (30–32). While CMC, endocrinopathies and non-endocrine manifestations such as pernicious anemia, nail dystrophy, vitiligo and alopecia have been reported, autoimmune pneumonitis has thus far not been reported in those carrying AD mutations in *AIRE*. The enrichment of the c.967_979del13 mutation in American and British cohorts may explain the differences in prevalence among Americans and British. Alternatively, or in parallel, non-*AIRE* genetic modifiers (33), differential pulmonary microbiome, environmental factors, and/or our unbiased enrollment coupled with a uniform prospective evaluation in all patients regardless of symptoms may contribute to the increased prevalence of pneumonitis among Americans. Future enrollment and uniform multidisciplinary evaluation of European and additional American patients in our and other institutions will be essential to validate our findings.

#### Thymic Escape of Autoreactive Lymphocytes

AIRE is expressed in thymic medullary epithelial cells (mTECs) where it facilitates the negative selection of self-reactive T-lymphocytes. As a transcription regulator, AIRE promotes the expression of peripheral tissue-restricted antigens on mTECs and the clonal deletion of self-reactive T-lymphocytes; in the AIRE-deficient state, these cells escape in the periphery and are both necessary and sufficient to cause tissue-specific autoimmunity as shown by lymphocyte depletion and adoptive transfer experiments in mice (2, 34–38).

AIRE-deficiency also impairs B-lymphocyte tolerance (39), which contributes to the development of autoimmunity in some, but not all, tissues (40). AIRE-deficient humans and mice produce a broad repertoire of high-affinity autoantibodies (1, 41–44), although these autoantibodies have not demonstrated direct pathogenicity *via* serum transfer studies in mice (37, 40). Instead, B-lymphocytes appear to contribute to autoimmune inflammation through priming effector T-lymphocytes (40).

Several tissue-specific autoantibodies correlate with the development of organ-specific disease in APECED (38, 45–47). Among these, autoantibodies against bactericidal/permeability-increasing fold-containing family B member 1 (BPIFB1) and the potassium channel regulator KCNRG have been associated with development of APECED pneumonitis (3, 21, 27, 48). We corroborated this finding in our cohort where both autoantibodies were highly specific for pneumonitis and significantly associated with the time to development of pneumonitis (5). Autoantibodies against BPIFB1 were more sensitive compared to those against KCNRG (5). Although the majority (76%) of affected patients carried at least one of these lung-targeted autoantibodies in serum and/or bronchoalveolar lavage (BAL), a quarter of patients with pneumonitis were negative for both autoantibodies. Therefore, while identification of autoantibodies in patient serum may aid as a screening modality of pneumonitis, such testing alone does not suffice to rule out pneumonitis in all individuals, further underscoring the importance of universal screening *via* chest CT imaging. Importantly, these data also underscore the need for future research aimed to identify the lung autoantigens that might be the target of autoimmune attack in patients with APECED pneumonitis who do not carry BPIFB1 or KCNRG autoantibodies.

#### Autoimmune-Polyendocrinopathy-Candidiasis-Ectodermal Dystrophy Pneumonitis Features a Characteristic Compartmentalized Immunopathology

**Figure 2:**
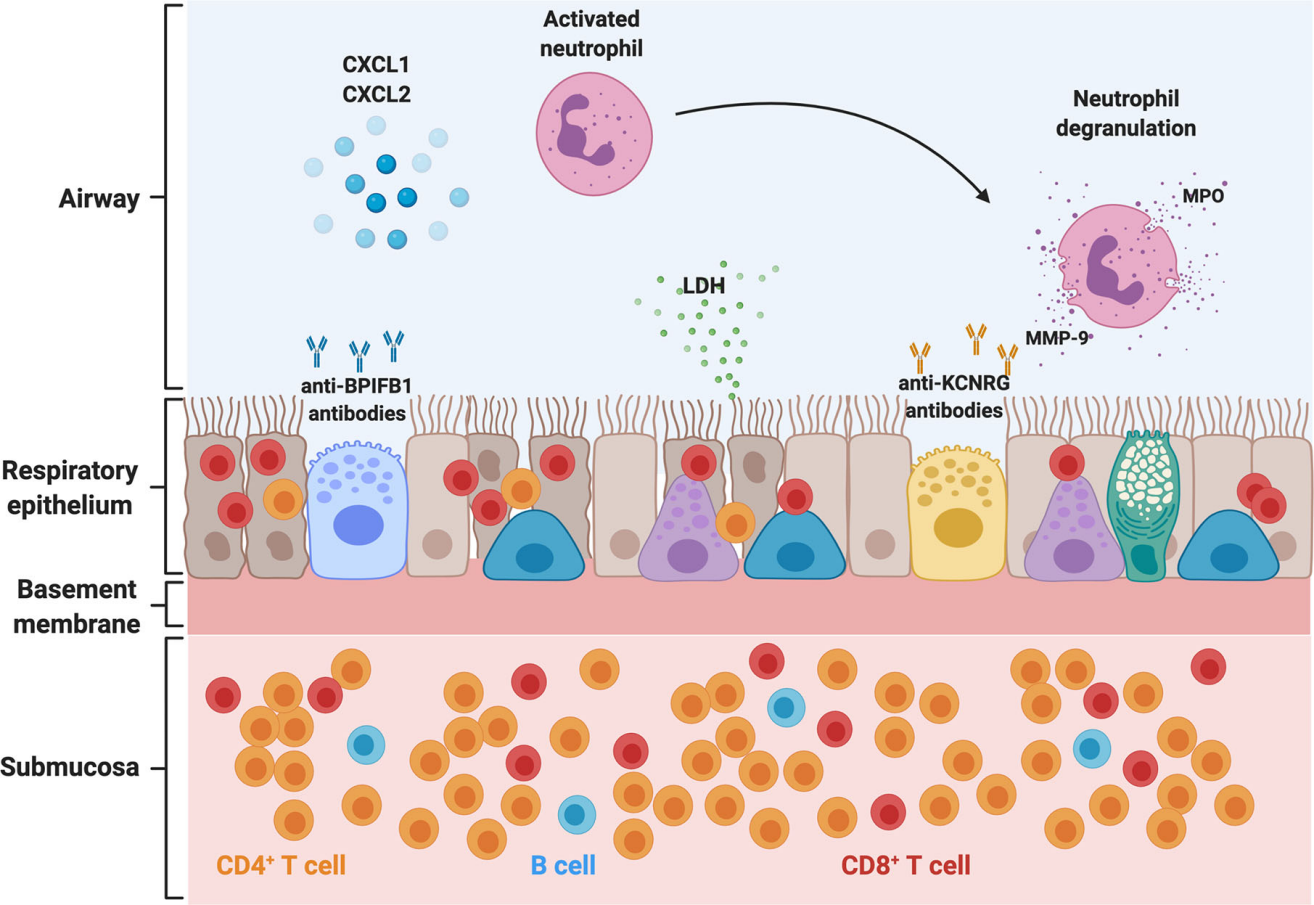
Pathogenesis of APECED-associated pneumonitis. Schematic representation of the abnormalities in the airway, respiratory epithelium, and submucosal tissue in the setting of APECED pneumonitis. T- and B-lymphocytes infiltrate the respiratory tissue. CD4^+^ T-lymphocytes predominate in the submucosal tissue and peribronchiolar/bronchiolar areas (not depicted), while CD8^+^ T-lymphocytes display a predominantly intraepithelial distribution. Neutrophils predominate in the airways where they accumulate through the release of CXC chemokines such as CXCL1, CXCL2, and IL-8. Recruited neutrophils acquire an activated phenotype and release MPO and MMP-9 into the airway, which further exacerbates tissue injury, as seen with release of LDH within the airways. Chronic epithelial irritation results in a thickened basement membrane. KCNRG and the BPIFB1 have been identified as bronchial autoantigens targeted by autoimmunity in APECED pneumonitis, and autoantibodies against these targets can be detected in the bronchoalveolar lavage and serum (not depicted) of patients with APECED pneumonitis. BPIFB1, bactericidal/permeability-increasing fold containing family B member 1; LDH, lactate dehydrogenase; MPO, myeloperoxidase; MMP-9, matrix metallopeptidase 9; CXCL1, C-X-C chemokine ligand 1; CXCL2, C-X-C chemokine ligand 2.

We performed bronchoscopies in APECED patients with untreated pneumonitis and obtained BAL fluid and endobronchial and transbronchial tissue biopsies for immunological and histological analyses in comparison to healthy volunteer specimens obtained in bronchoscopy. A characteristic compartmentalized immune response was noted, which carries significant diagnostic value. In the airways, an enrichment of neutrophils was seen in the absence of bacterial or other lung infection. In agreement, we observed a significant increase of neutrophil-targeted CXC chemokines in the BAL (CXCL1, CXCL2, IL-8), although the cellular source of these chemokines remains unknown (**Figure 2**). BAL neutrophils exhibited an activated phenotype evidenced by increased expression of the extracellular epitope of the NADPH oxidase b558, of primary, secondary, and tertiary granule contents (CD18, CD63, CD66b), and of CD45, and decreased CD16 expression. Both myeloperoxidase (MPO) and matrix metallopeptidase-9 (MMP-9), products of activated neutrophils, and lactate dehydrogenase (LDH), a surrogate marker of tissue injury, were markedly increased in the BAL fluid of patients with pneumonitis (5). Thus, activated neutrophils appear to contribute to airway tissue injury and may instigate bronchiectasis as postulated in patients with cystic fibrosis and non-cystic fibrosis bronchiectasis (49, 50).

In contrast to the neutrophilic response in the airways, histological examination of endobronchial and deeper lung tissue biopsies demonstrated a chronic inflammatory infiltrate consistent with prior literature describing lymphocytic peribronchiolar inflammation in few patients (**Figure 2**) (5, 26, 27, 51). Endobronchial biopsies from patients with APECED pneumonitis displayed a thickened basement membrane with submucosal and intraepithelial lymphocytosis composed predominately of T-lymphocytes with fewer B-lymphocytes. CD4^+^ T-lymphocytes predominated in the submucosa whereas CD8^+^ T-lymphocytes were enriched within the intraepithelial compartment (**Figure 2**) (5). No eosinophils or neutrophils were observed infiltrating the tissue. Deeper lung biopsies unveiled lymphocytic or lymphoplasmacytic bronchiolitis and/or peribronchiolar inflammation dominated by CD4^+^ and CD8^+^ T-lymphocytes, with mild-to-moderate fibrosis noted in some patients. As with endobronchial biopsy specimens, CD8^+^ T-lymphocytes predominated within the bronchiolar epithelium while CD4^+^ T-lymphocytes were prominent in the submucosal bronchiolar tissue. Notably, whereas infiltration of B-lymphocytes was less prominent on endobronchial biopsy specimens, deep peribronchial tissue examination demonstrated marked B-lymphocyte infiltration with development of lymphoid nodules and primary follicles, some of which showed germinal center formation (5).

The mouse model of Aire-deficiency recapitulated the immunological characteristics of autoimmune pneumonitis of patients. Specifically, *Aire* ^-/-^ mice exhibited airway neutrophilia with increased neutrophil-targeted CXC chemokines in the absence of an infectious challenge. Moreover, the lung parenchyma of *Aire* ^-/-^ mice featured similar histological abnormalities consisting of intraepithelial, submucosal, peribronchiolar and interstitial infiltration composed of T- and B-lymphocytes with B-lymphocyte aggregates observed deeper in the lung tissue (5).

Collectively, APECED pneumonitis features a characteristic pattern of compartmentalized immunopathology consisting of activated neutrophils in the airways with lymphocytic inflammation within the lung parenchyma. This information has important diagnostic value. For example, the presence of neutrophils in the BAL or even in induced sputum examination in an APECED patient with pulmonary symptoms and radiographic abnormalities should raise suspicion for pneumonitis in the absence of pneumonia. Endobronchial biopsies, which we favor as the preferred modality for making a histological diagnosis of pneumonitis, allow for demonstration of intraepithelial and submucosal lymphocytosis, which together with the airway neutrophil expansion provide a high degree of probability for the diagnosis of APECED pneumonitis, especially when combined with BPIFB1- and/or KCNRG-targeted autoantibody positivity.

## Combination Lymphocyte-Directed Immunomodulation Remits Pneumonitis

Previous reports of various immunomodulatory treatments had demonstrated mixed results with one patient responding to T-lymphocyte immunomodulation with azathioprine (27) while other patients required multiple different T-lymphocyte therapies with mixed results (21, 27). Data in the Aire-deficient mouse from our group and others would suggest that a T-lymphocyte depletion approach such as with the CD52-targeting alemtuzumab would remit APECED pneumonitis (5, 37); however, the risk of opportunistic infections makes such T-cell depleting strategies difficult to implement for the lifelong management of pneumonitis (52, 53). Thus, we elected a combination of T-lymphocyte modulation with azathioprine [or mycophenolate mofetil in patients with thiopurine methyltransferase (TPMT) mutations] together with B cell-targeting rituximab to capitalize on the beneficial effects of B-lymphocyte deficiency observed in mice (5). This regimen is used successfully to treat granulomatous and lymphocytic interstitial lung disease (GLILD) seen in combined variable immunodeficiency (CVID) (54).

Combination T and B lymphocyte-directed therapy resulted in resolution of respiratory symptoms in all symptomatic patients within 1 month. Those who had recurrent pulmonary infections secondary to their bronchiectasis before onset of immunomodulatory treatment did not develop infection recurrences after therapy initiation, indicating that the hyper-inflammatory milieu within the untreated airways is permissive for pathogen overgrowth. Immunomodulatory treatment was accompanied by marked improvement of radiographic abnormalities of GGO, tree-in-bud pattern, nodular opacities, and mucus plugging. Improvement was also noted in pulmonary function abnormalities with increased 6 min walk distance and resolution of oxygen desaturation (5). Lymphocyte immunophenotyping showed no changes in CD3^+^, CD4^+^, and CD8^+^ T-lymphocyte numbers in blood and an expected decline in CD19^+^ B-lymphocytes. Titers of BPIFB1 and KCNRG autoantibodies did not decline despite clinical and radiographic remission of pneumonitis, further suggesting that the pathogenic role of B-cells might be conferred *via* priming of T-cells in the lung tissue, rather than through autoantibody production. This early treatment study of five consecutive patients (5) with pneumonitis and treatment of 6 additional patients with similar results (manuscript in preparation) indicate that combination T and B lymphocyte-directed therapy can remit clinical symptoms and radiographic and functional abnormalities in APECED pneumonitis. Importantly, early initiation of treatment, preferably before the establishment of irreversible bronchiectatic abnormalities, is desirable to avoid the long-term pulmonary complications and morbidity and mortality associated with untreated pneumonitis.

## Autoimmune-Polyendocrinopathy-Candidiasis-Ectodermal Dystrophy Pneumonitis Shares Immunological Features With Interstitial Lung Diseases Associated With Secondary Autoimmune Regulator-Deficiency States

Conditions associated with documented secondary AIRE-deficiency in the thymus such as thymoma (55) and inherited RAG deficiency due to hypomorphic *RAG* mutations that cause delayed onset combined immunodeficiency with granulomas and/or autoimmunity (CID-G/AI) feature autoimmunity and display broad-spectrum autoantibodies against cytokines and tissue autoantigens (56–58) similar to APECED patients. A subset of these patients develops lung disease, which had previously been poorly-characterized (59, 60). We hypothesized that the lung disease seen in patients with thymoma or hypomorphic *RAG* mutations share similar features with APECED pneumonitis. Indeed, thymoma-associated autoimmune lung disease exhibits a similar compartmentalized immunopathology with airway neutrophil expansion and intraepithelial, submucosal, and peri-bronchiolar lymphocytic inflammation as seen in APECED pneumonitis (5). A smaller proportion of these patients carry autoantibodies against BPIFB1 and KCNRG compared to patients with APECED pneumonitis (5), pointing to additional yet-unidentified lung autoantigens in these diseases. Notably, the similarities between autoimmune lung disease seen in the setting of these secondary AIRE-deficiency states and APECED suggest common pathogenetic mechanisms and imply that the lymphocyte-targeted immunomodulatory regimen that is effective in APECED pneumonitis might also remit ILD in patients with thymoma (manuscript in preparation) and may serve as a bridge to hematopoietic stem cell transplantation in patients with ILD in the setting of hypomorphic RAG mutations with CID-G/AI.

Beyond primary and secondary AIRE-deficiency states, ILD with a similar compartmentalized immunopathology consisting of airway neutrophil expansion and lymphocytic bronchiolitis develops among a subset of patients with certain polygenic autoimmune diseases such as Sjögren’s syndrome (SS), ulcerative colitis (UC), systemic lupus erythematosus (SLE), and dermatomyositis (DM) (61–64). Future research is required to determine whether, based on the shared pathologic features of these ILDs with APECED pneumonitis, these ILDs may also be responsive to the lymphocyte-directed therapy that is effective in APECED pneumonitis and GLILD. In addition, whether other primary immune dysregulatory disorders that manifest with ILD such as STAT3 gain-of-function (GOF), CTLA4 haploinsufficiency, and LRBA deficiency share common immunopathological mechanisms with APECED pneumonitis merits future investigation (65–68).

## Conclusion

Herein, we highlighted clinical, radiographic, pulmonary function, autoantibody, immunological, and histological abnormalities of APECED pneumonitis, a previously-unrecognized manifestation of AIRE-deficiency that causes significant morbidity and mortality when untreated. Periodic screening with chest CT and bronchoscopic performance of endobronchial biopsies to reveal the characteristic compartmentalized immunopathology of pneumonitis have important implications for early diagnosis and initiation of lymphocyte-directed immunomodulation that can remit pneumonitis and prevent irreversible pulmonary complications. The common immunological and histological features between APECED pneumonitis and ILDs seen in secondary AIRE-deficiency states (thymoma, RAG deficiency), and certain polygenic autoimmune disorders (SS, UC, SLE, DM) suggest that the pathogenesis of autoimmune lung disease is shared among disorders of central immune tolerance and show promise for the potential efficacy of a similar lymphocyte-directed immunomodulatory regimen for these common ILDs.

## Author Contributions

EF conducted the literature review and wrote the initial draft of the manuscript. ML revised the manuscript. All authors contributed to the article and approved the submitted version.

## Funding

This research was supported by the Division of Intramural Research of the National Institute of Allergy and Infectious Diseases, National Institutes of Health, USA.

## Conflict of Interest

The authors declare that the research was conducted in the absence of any commercial or financial relationships that could be construed as a potential conflict of interest.
